# Comparison of expression profiles between undifferentiated and differentiated porcine IPEC-J2 cells

**DOI:** 10.1186/s40813-022-00247-0

**Published:** 2022-01-09

**Authors:** Guolin Pi, Wenxin Song, Zijuan Wu, Yali Li, Huansheng Yang

**Affiliations:** Hunan Provincial Key Laboratory of Animal Intestinal Function and Regulation, Hunan International Joint Laboratory of Animal Intestinal Ecology and Health, Hunan Normal University, No. 36 Lushan Road, Changsha, 410081 Hunan China

**Keywords:** IPEC-J2 cells, Cellular differentiation, Apoptosis, Gene expression

## Abstract

**Background:**

The intestinal porcine enterocyte cell line (IPEC-J2) is a well-established model to study porcine intestinal physiology. IPEC-J2 cells undergo spontaneous differentiation during culture while changes in expression patterns of differentiated IPEC-J2 remain unclear. Therefore, this study was aimed to investigate the expression profiles of IPEC-J2 cells at the transcriptional level. Differentially expressed genes (DEGs), enriched pathways and potential key genes were identified. Alkaline phosphatase (AKP) and percentages of apoptotic cells were also measured.

**Results:**

Overall, a total of 988 DEGs were identified, including 704 up-regulated and 284 down-regulated genes. GO analysis revealed that epithelial cell differentiation, apoptotic signaling pathway, regulation of cytokine production and immune system process, regulation of cell death and proliferation, cell junction complexes, and kinase binding were enriched significantly. Consistently, KEGG, REACTOME, and CORUM analysis indicated that cytokine responses modulation may be involved in IPEC-J2 differentiation. Moreover, AKP activity, a recognized marker of enterocyte differentiation, was significantly increased in IPEC-J2 after 14 days of culture. Meanwhile, annexin V-FITC/PI assay demonstrated a remarkable increase in apoptotic cells after 14 days of culture. Additionally, 10 hub genes were extracted, and STAT1, AKT3, and VEGFA were speculated to play roles in IPEC-J2 differentiation.

**Conclusions:**

These findings may contribute to the molecular characterization of IPEC-J2, and may progress the understanding of cellular differentiation of swine intestinal epithelium.

## Background

The intestinal porcine enterocyte cell line (IPEC-J2) is a non-transformed, permanent intestinal cell line, originally derived from the jejunum of a neonatal unsuckled piglet [1, 2]. IPEC-J2 cells exhibit strong similarities to primary intestinal epithelial cells, and serve as a well-established model to study effects of nutrients and other feedstuffs in the gut prior to in vivo evaluation. Numerous studies have used this cell line to investigate the influence of extracellular amino acids [2], fatty acids [3], vitamins [4], trace elements [5] and other nutrients [6, 7] on epithelial cell metabolism and function. Besides its epithelial nature, IPEC-J2 cells also provide an ideal tool for studying the interactions between porcine intestinal epithelial cell and enteric bacteria [8, 9]. Moreover, IPEC-J2 cells more closely mimic the human physiology than any other cell line of non-human origin. Hussain et al. [10] used IPEC-J2 as *in-vitro* model to evaluate the cytoprotective effects of natural bioactive compounds against LPS mediated inflammatory response, due to their higher sensitivity to LPS stimulation than Caco-2 cells. Thus, IPEC-J2 cells provide a valuable research tool to improve the understanding of nutrition science, microbial pathogenesis, immune processes, and intestinal disorders in both animals and humans.

Enterocyte cells differentiate during their migration along the crypt-villus axis [11]. Differentiated enterocytes then undergo a process of apoptosis and are extruded into the lumen [12, 13]. IPEC-J2 cells maintain their differentiated characteristics and undergo a process of spontaneous differentiation leading to the formation of a polarized monolayer [1]. Schierack et al. [8] reported that IPEC-J2 formed largely single cell monolayers on day 14 of culture. Electron microscopy showed apical microvilli of differing lengths and widths, and immunostaining revealed tight junction proteins at the apicolateral membrane [8]. Another study by Geens and Niewold optimized the culture conditions of IPEC-J2 and revealed that, well formed apical microvilli were present from day 9 onwards [14]. Moreover, the authors examined the progression in trans-epithelial electrical resistance (TEER) of cell monolayer, and they found that IPEC-J2 cells grown on collagen-coated membranes reached a resistance maximum after 16 days of culture [14]. The differentiated cells can be used to investigate the impact of compounds on the monolayer permeability or tight junction structures. So far, IPEC-J2 cells have been characterized morphologically and functionally while few studies have been carried out on molecular level. Changes in expression patterns of undifferentiated and differentiated IPEC-J2 monolayer remain unclear.

In this study, we intended to investigate the expression profiles of differentiated IPEC-J2 cells at the transcriptional level. Differentially expressed genes (DEGs), enriched pathways and potential key genes were identified, and the cell differentiation and apoptosis were also examined. This study may provide a theoretical basis for the molecular characterization of IPEC-J2 cells, which can be of great interest for intestinal research.

## Materials and methods

### Cell culture

IPEC-J2 cells were kindly supplied by Dr Yin Yulong’s laboratory (Institute of Subtropical Agriculture, Chinese Academy of Sciences). Cells were used between passages 70 and 90, and were seeded at 2 × 10^5^ cells/mL in 6-well plates. Cells were cultured in DMEM/F12 supplemented with 10% FBS (Invitrogen, Carlsbad, US), 100 UI/mL penicillin, and 100 μg/mL streptomycin at 37 °C in a humidified atmosphere with 5% CO_2_. The medium was changed three times weekly, according to standard culture protocols [3, 8].

### RNA extraction and microarray data analysis

IPEC-J2 cells were seeded at 2 × 10^5^ cells/mL in 6-well plates and cultured for 14 days and then lysed with TRIZOL Reagent (Life technologies, Carlsbad, USA). Isolation of total RNA was performed following the manufacturer’s instructions and checked for purity and concentration. High-quality RNA (RIN value > 9.0) was used for microarray examination by the Illumina NovaSeq6000 platform. Differentially expressed genes (DEGs) were identified based on the cut-off criteria (Probability ≥ 0.8 and |log2(fold change)|≥ 1). Metascape (https://metascape.org) was used to perform functional enrichment analysis [15]. Gene Ontology (GO), Kyoto Encyclopedia of Genes and Genomes (KEGG), Reactome, and Comprehensive Resource of Mammalian protein complexes (CORUM) was carried out to expound promising signaling pathways correlated with DEGs. Interaction network was constructed and visualized using Cytoscape, and nodes represented enriched terms colored by cluster and P-value. The CytoHubba plugin of Cytoscape was used to determine hub genes in the network. The top 10 hub genes were selected with the maximal clique centrality (MCC) method.

### Measurements of alkaline phosphatase activity

Alkaline phosphatase (AKP) in IPEC-J2 cells was measured by AKP assay kit (Beyotime; P0321S) according to the manufacturer’s instructions. Briefly, IPEC-J2 cells were seeded at a density of approximately 2 × 10^5^ cells/mL in triplicate in 6-well plates. Cells were then lysed after 1, 7, or 14 days of culture. Lysates were incubated with para-nitrophenyl phosphate in AKP-buffer for 10 min at 37 °C, and the substrate turnover (p-nitrophenol) was measured by spectrophotometer at 405 nm. Cellular protein concentrations were determined using the BCA method (Pierce). AKP activity was calculated in units/g protein (U/g protein).

### Apoptosis detection

Progression of IPEC-J2 cells apoptosis was assessed after 1, 7, and 14 days of culturing. In brief, IPEC-J2 cells were seeded at a 2 × 10^5^ cells/mL density in triplicate in 6-well plates. To detect apoptosis, cells were harvested and centrifuged at 3000 rpm for 3 min. Cells were stained using an Annexin V-FITC and propidium iodide (PI) apoptosis kit (eBioscience, San Diego) according to the manufacturer’s protocol. After 15 min incubation under dark conditions, stained cells were subjected immediately to flow cytometry analysis with an Accuri C6 FACS machine. Viable cells remained unstained (Annexin V−/PI−). Early apoptotic cells exhibited Annexin V+/PI-staining patterns; while late apoptotic cells were visualized with Annexin V-FITC+/PI + staining patterns. Experiments were repeated three times.

### Statistical analysis

Functional enrichment analysis was examined by Metascape utilizing the hypergeometric test and Benjamini–Hochberg *P*-value correction algorithm. Pairwise similarities between two enriched terms were computed based on a Kappa-test score, and enrichment networks were created with Kappa similarities above 0.3. Hub genes were selected with the maximal clique centrality (MCC) method. Results of AKP activity are presented as the mean ± SEM, and statistical significance was determined using one‐way ANOVA followed by Tukey’s post hoc analysis with GraphPad Prism (GraphPad Software, San Diego, CA). Significance was defined as a *P*-value < 0.05.

## Results

### Identification of differentially expressed mRNAs in IPEC-J2 cells

**Fig. 1:**
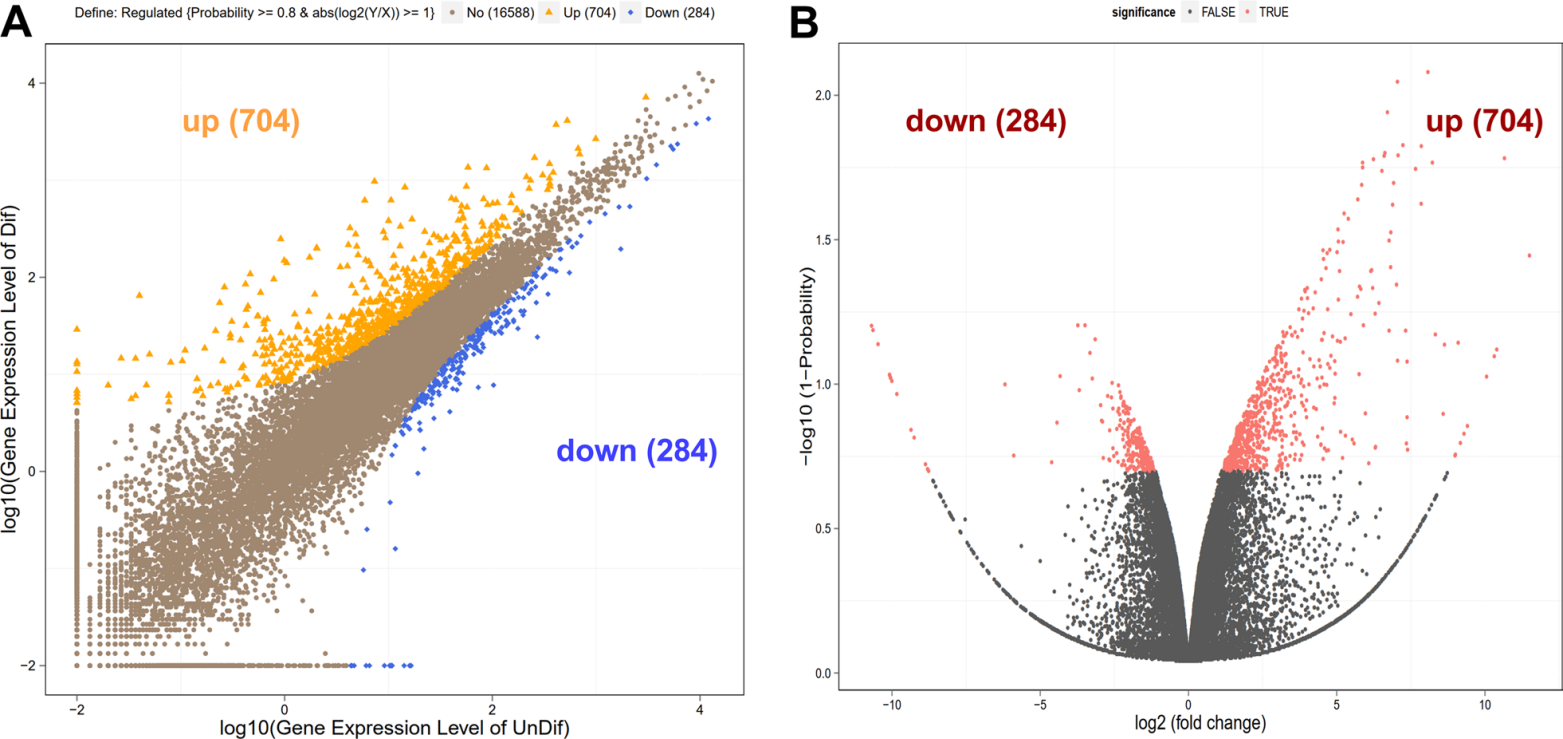
Differentially expressed genes (DEGs) in IPEC-J2 cells. DEGs between differentiated and undifferentiated IPEC-J2 cells were visualized by the scatter plot (**A**) and volcano plot (**B**). DEGs were identified based on the cut-off criteria (probability ≥ 0.8 and |log2(fold change)|≥ 1)

Differentially expressed genes (DEGs) between differentiated and undifferentiated IPEC-J2 cells were visualized by the scatter plot (Fig. 1A) and volcano plot (Fig. 1B). A total of 17,576 genes were obtained from the original RNA-seq data. Based the cut-off criteria (Probability ≥ 0.8 and |log2(fold change)|≥ 1), a total of 988 differentially expressed mRNAs were identified, including 704 up-regulated and 284 down-regulated genes in IPEC-J2 cells (Fig. 1A, B).

### Gene ontology analysis

**Fig. 2:**
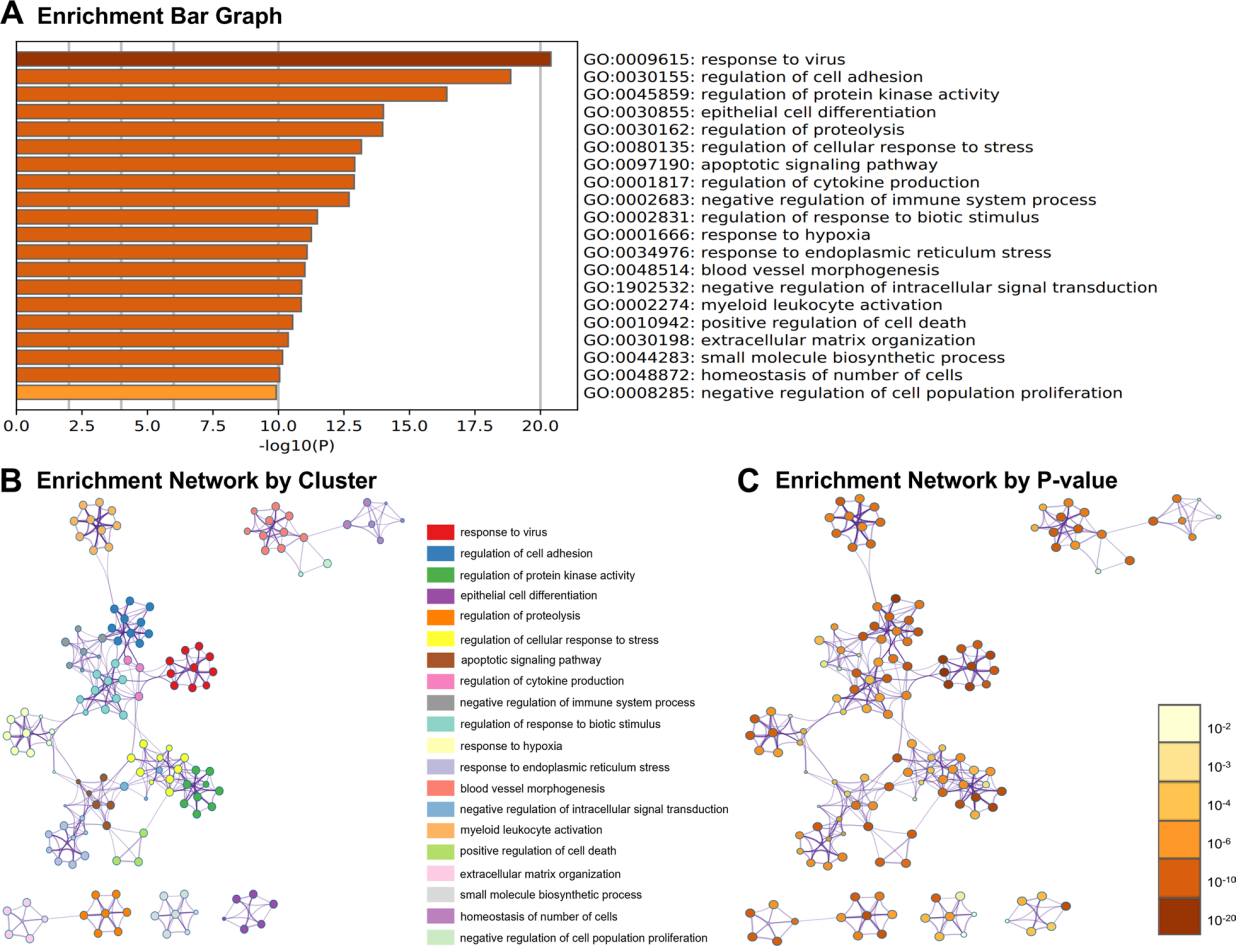
The enriched Gene Ontology (GO) terms in biological process (BP). The top 20 enriched GO terms in BP (**A**). Interaction network was constructed with Kappa similarities above 0.3, and visualized using Cytoscape, and nodes represented enriched terms colored by cluster (**B**) and *P*-value (**C**). A circle node represented each term. Node size was proportional to the number of genes that fell into that term, while node color represented the identity of the cluster to which it belonged. Cluster annotations were shown in color code (**B**). Discrete color scale represented statistical significance. Darker colors indicated higher enrichment significance (**C**)

**Fig. 3:**
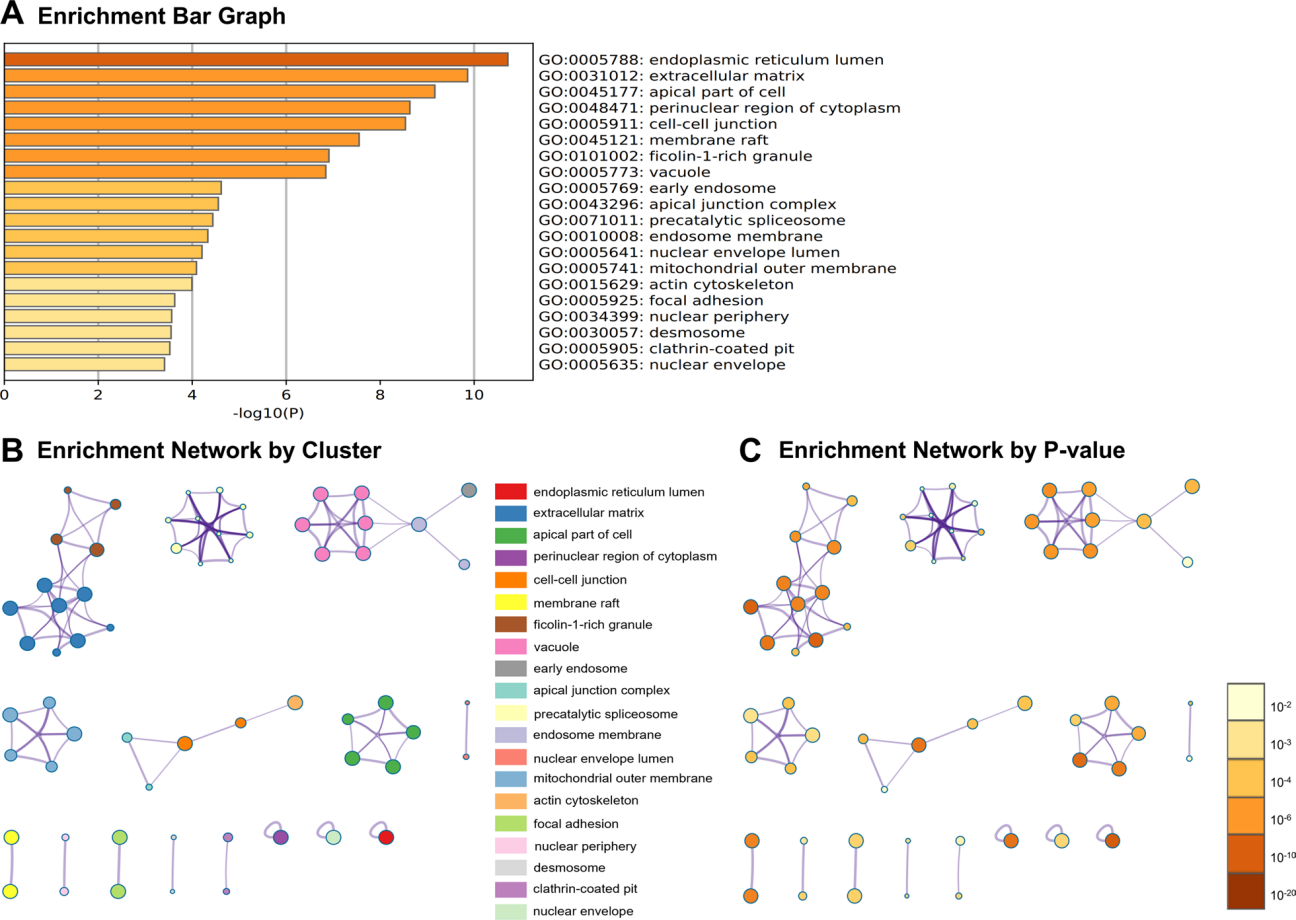
The enriched Gene Ontology (GO) terms in cellular component (CC). The top 20 enriched GO terms in CC (**A**). Network of enriched terms was shown in cluster (**B**) and *P*-value (**C**) form

**Fig. 4:**
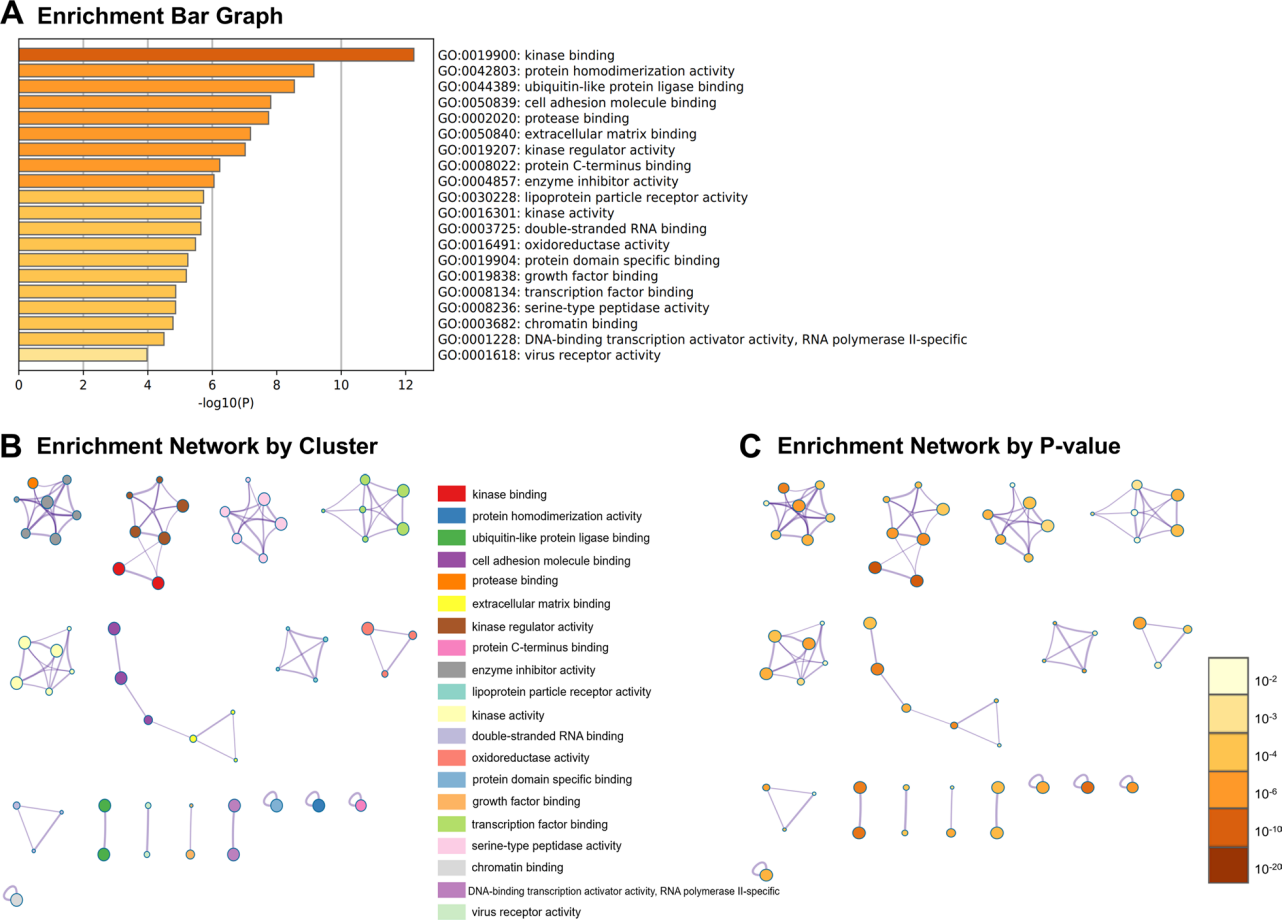
The enriched Gene Ontology (GO) terms in molecular function (MF). The top 20 enriched GO terms in MF (**A**). Interaction network was integrated by cluster (**B**) and *P*-value (**C**)

The top 20 enriched gene ontology (GO) biological process terms (BP) (Fig. 2), cell component terms (CC) (Fig. 3), and molecular function terms (MF) (Fig. 4) are shown according to the *P*-value. Our results showed that response to virus, regulation of cell adhesion, epithelial cell differentiation, apoptotic signaling pathway, regulation of cytokine production and immune system process, and regulation of cell death and proliferation, were significantly enriched in BP terms (Fig. 2A). Moreover, endoplasmic reticulum lumen, extracellular matrix, apical part of cell, cell–cell junction, apical junction complex, focal adhesion, and desmosome, were identified in CC terms (Fig. 3A). Further, kinase binding, protein homodimerization activity, ubiquitin-like protein ligase binding, cell adhesion molecule binding, protease binding, extracellular matrix binding, and kinase regulator activity, were enriched in MF terms (Fig. 4A). In addition, network of enriched BP, CC, and MF terms were integrated by cluster (Figs. 2, 3, and 4B) and *P*-value (Figs. 2, 3, and 4C). Network showed the intra-cluster and inter-cluster similarities of enriched terms. In Figs. 2, 3, and 4B, nodes sharing the same cluster were shown in the same color. Clusters were bridged through terms with similarities reflecting the relatedness of two separate processes. In Figs. 2, 3, and 4C, discrete color scale represented statistical significance. Terms have deeper color tend to have lower *P*-values.

### Pathway enrichment analysis

**Fig. 5:**
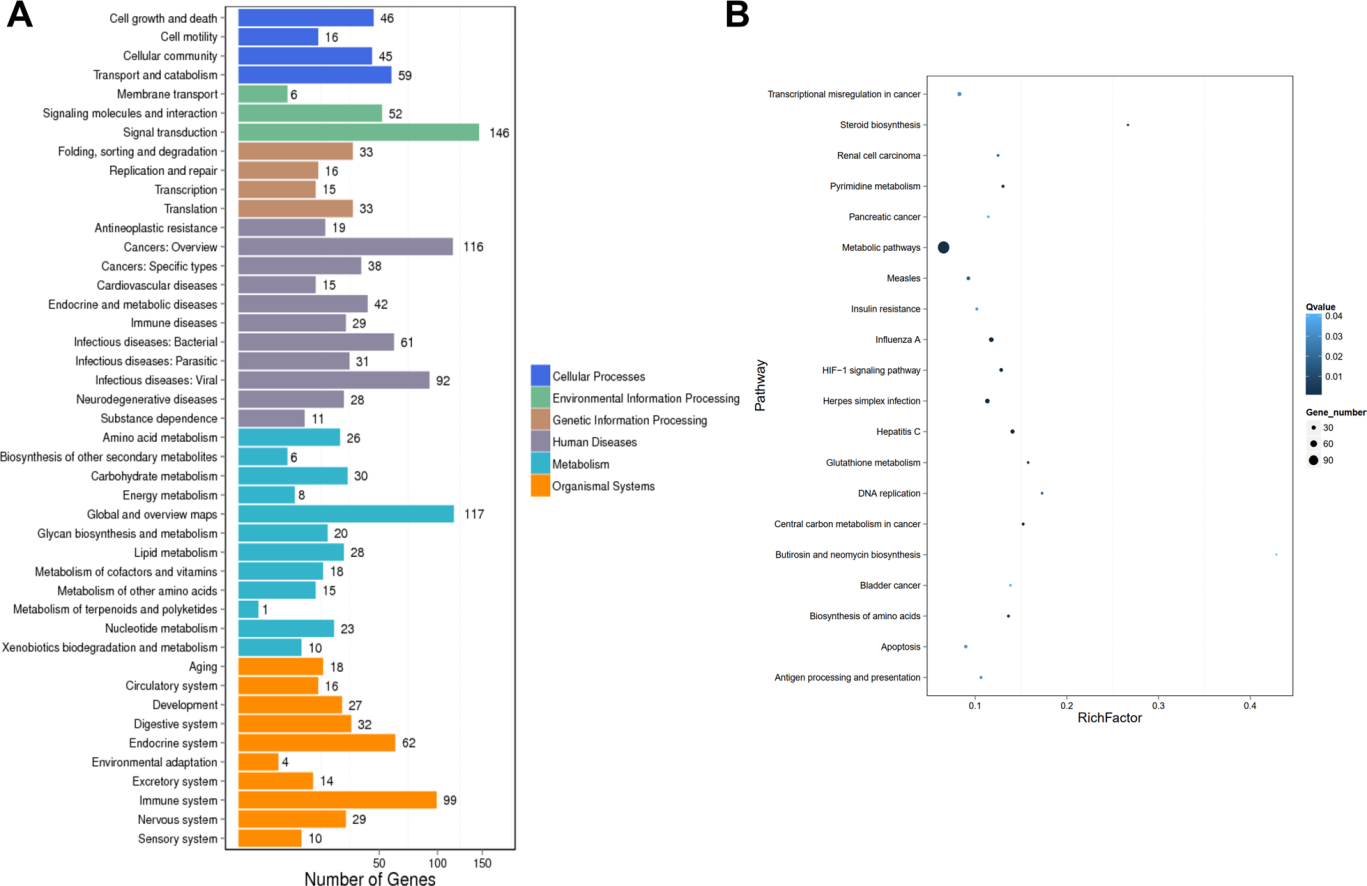
KEGG pathway enrichment analysis. Enriched terms of KEGG pathway were presented as histogram (**A**) and bubble charts (**B**). The vertical axis described the name of the pathway, and the horizontal axis showed the number of genes (**A**). The vertical axis represented various pathways, and the horizontal indicated the corresponding rich factor of the pathway. Higher rich factors suggested greater degrees of enrichment. The bubble size indicated the number of genes enriching the corresponding annotation, while the color represented statistical significance (**B**)

**Fig. 6:**
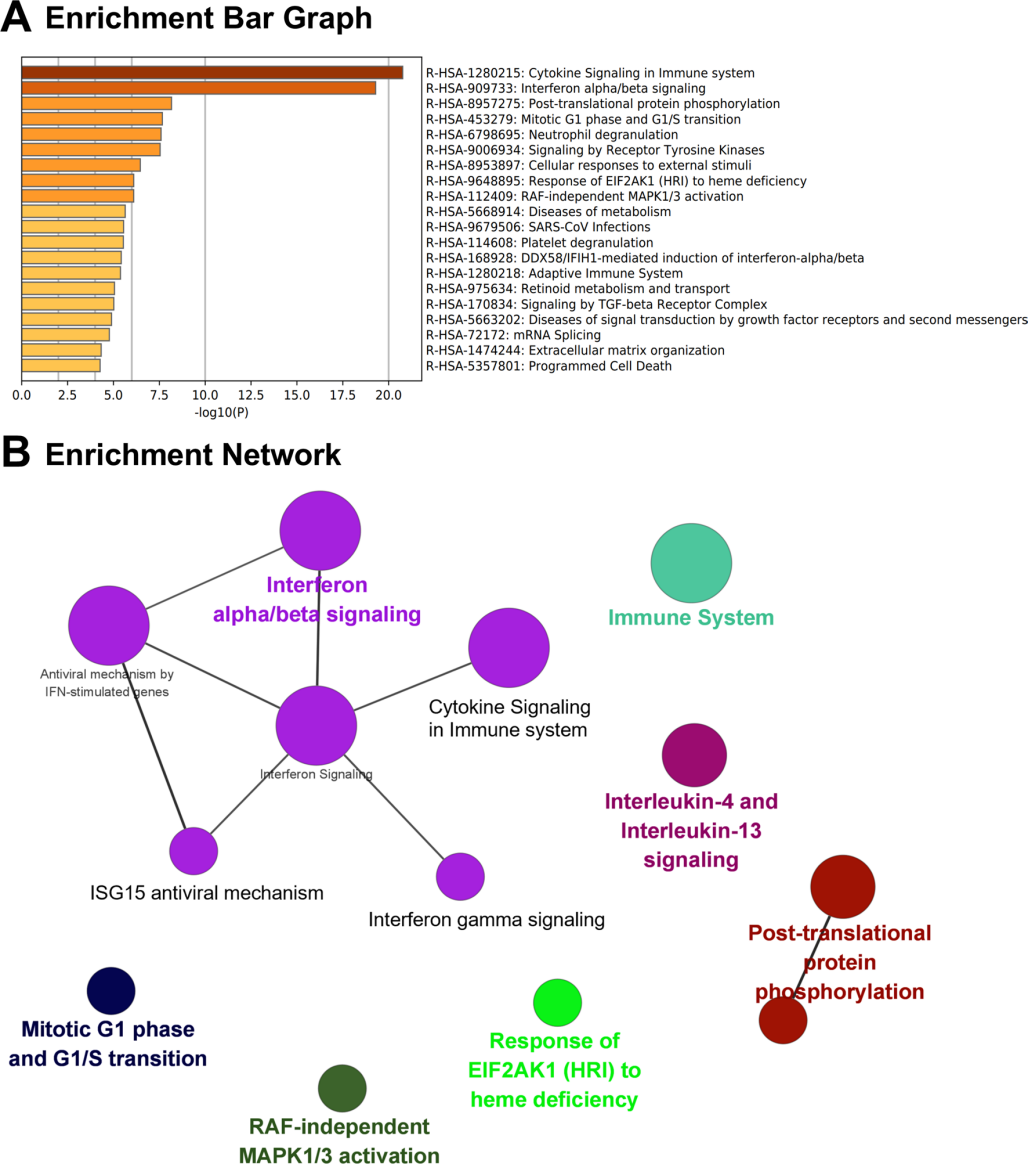
REACTOME enrichment analysis. The top 20 enriched terms were shown in (**A**). Interaction network was constructed and visualized using Cytoscape (**B**)

**Fig. 7:**
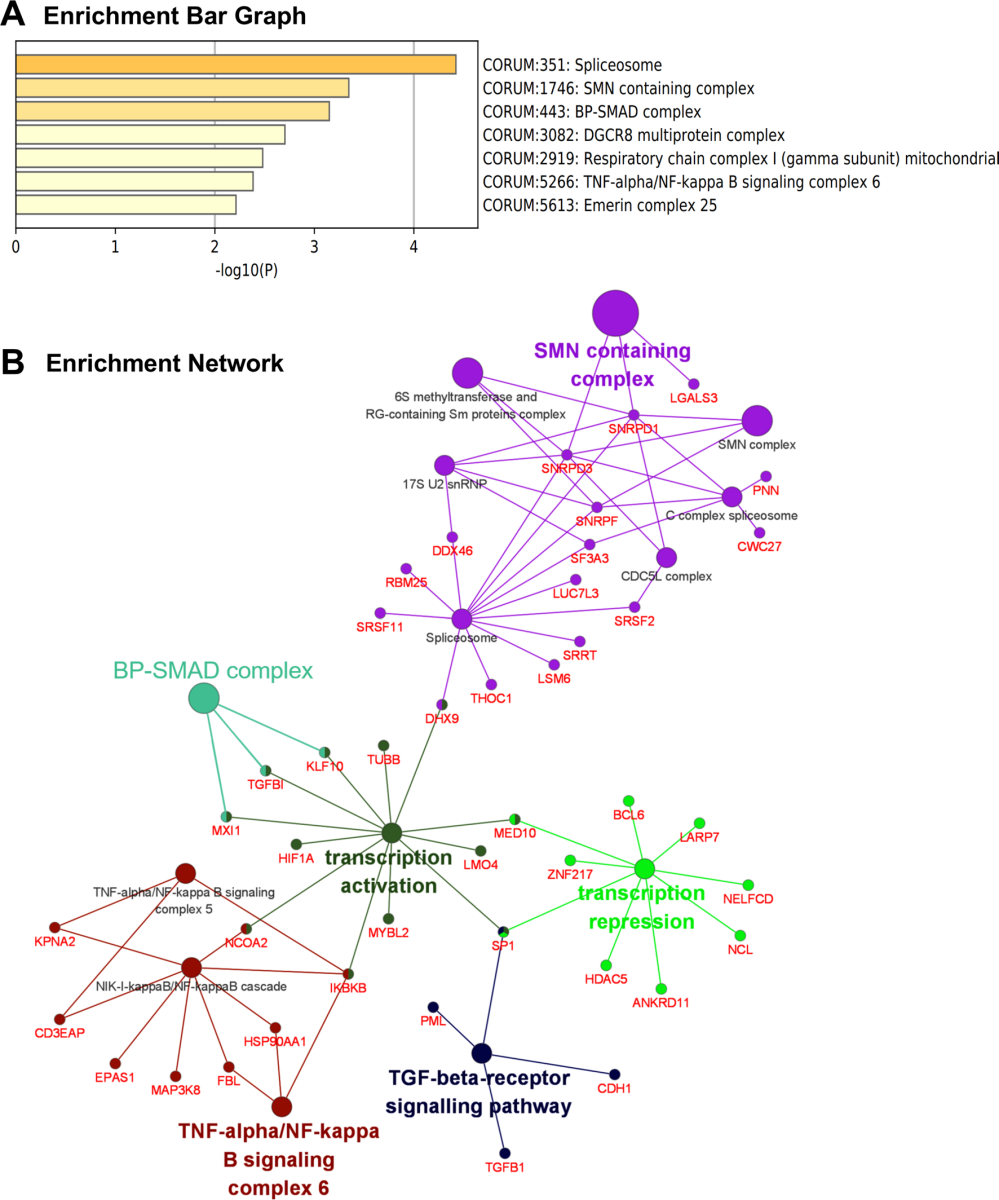
CORUM enrichment analysis. Enriched terms were demonstrated in (**A**). Interaction network was constructed and visualized using Cytoscape (**B**)

To clarify the involved biological regulation processes, gene set enrichment for KEGG, REACTOME, and CORUM pathway analysis was performed. According to KEGG analysis, DEGs were mainly involved in the signaling pathways associated with metabolism (metabolic pathways, steroid biosynthesis, pyrimidine metabolism, glutathione metabolism, and biosynthesis of amino acids), cancers, and immune system (Fig. 5A, B). Consistently, REACTOME analysis revealed that DEGs were mainly enriched in cytokine signaling in immune system (R-1280215, -909733, -168928, -1280218, -170834), post-translational protein phosphorylation (R-8957275) and also in the cell cycle (R-453279) (Fig. 6A, B). Further, enriched protein complex spliceosome, SMN containing complex, BP-SMAD complex, and TNF-alpha/NF-kappa B signaling complex 6 were identified by CORUM analysis (Fig. 7A) and the related genes were shown in Fig. 7B.

### Hub gene selection

**Fig. 8:**
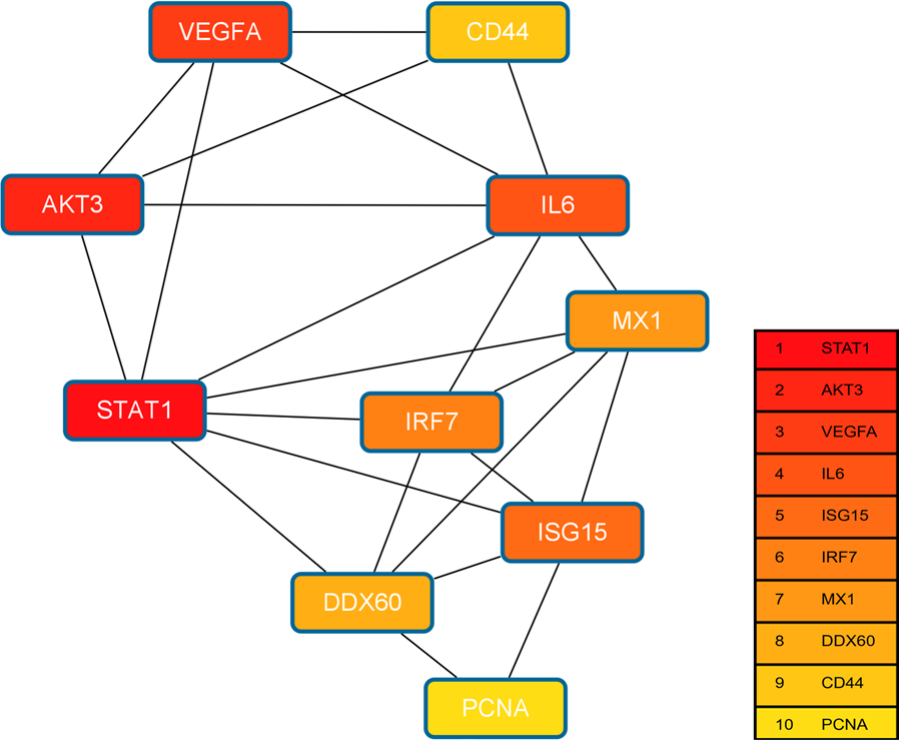
Network of 10 hub genes. Hub genes with high degree of connectivity were determined by the CytoHubba plugin of Cytoscape and selected with the maximal clique centrality (MCC) method

The top 10 hub genes with a high degree of connectivity, including Signal Transducer And Activator Of Transcription 1 (STAT1), AKT Serine/Threonine Kinase 3 (AKT3), Vascular Endothelial Growth Factor A (VEGFA), Interleukin 6 (IL-6), ISG15 Ubiquitin Like Modifier (ISG15), Interferon Regulatory Factor 7 (IRF7), MX Dynamin Like GTPase 1 (MX1), DExD/H-Box Helicase 60 (DDX60), CD44 Molecule (CD44), and Proliferating Cell Nuclear Antigen (PCNA), were determined by CytoHubba and demonstrated in Fig. 8.

### Cell differentiation analysis

**Fig. 9:**
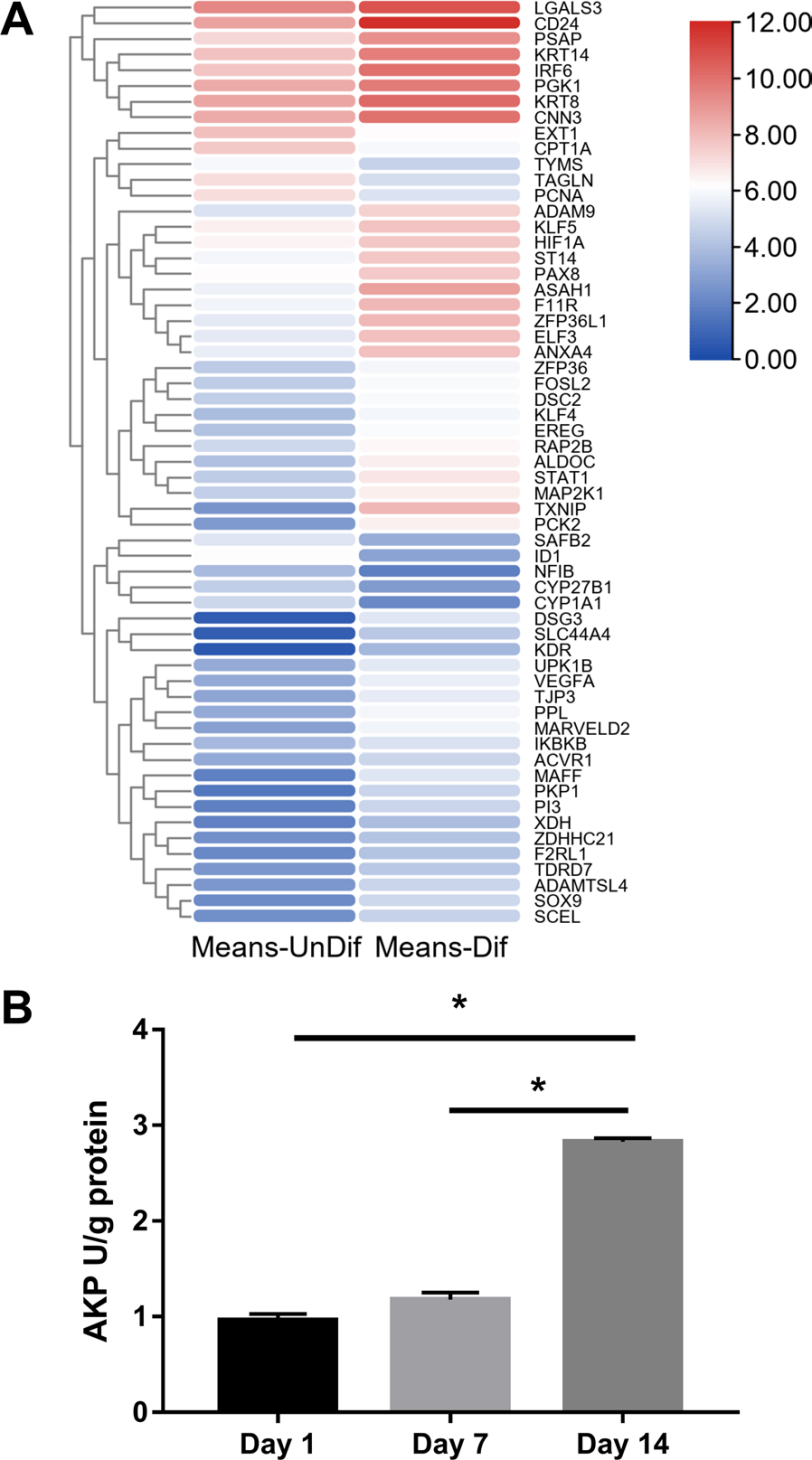
Cell differentiation analysis. Heatmap of differentiation-related genes (**A**) and alkaline phosphatase (AKP) activity in IPEC-J2 cells (**B**). Values for AKP was expressed as units/g protein (U/g protein)

By comparing expression data between differentiated IPEC-J2 cells and undifferentiated cells, a total of 59 differentiation-related genes were identified, including 49 up-regulated and 10 down-regulated genes (Fig. 9A). Moreover, alkaline phosphatase activity (AKP) in the cell lysate was measured to assess differentiation over time. As shown in Fig. 9B, significantly increased levels of AKP activity in IPEC-J2 cells were observed after 14 days of culture.

### Apoptotic analysis

**Fig. 10:**
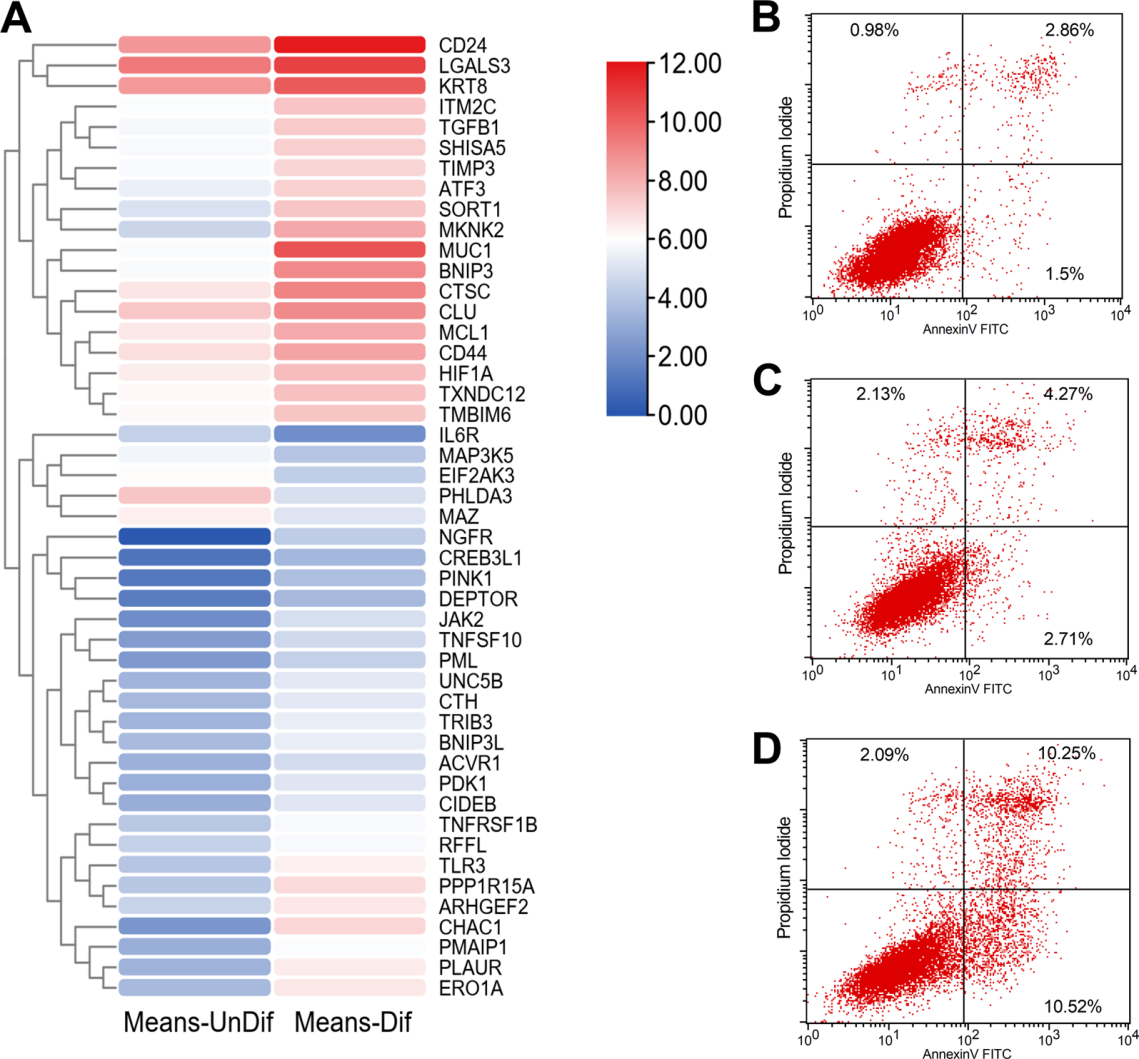
Apoptotic analysis. Heatmap of DEGs associated with apoptosis (**A**). Flow cytometric analysis for Annexin V-FITC/PI staining (**B**–**D**). Viable cells remained unstained (Annexin V−/PI−). Early apoptotic cells exhibited Annexin V+/PI-staining patterns; while late apoptotic cells were visualized with Annexin V-FITC+/PI+ staining patterns. Representative of at least three experiments

Heatmap of differentially expressed genes associated with apoptosis in differentiated IPEC-J2 cells compared to undifferentiated cells was demonstrated in Fig. 10A. A total of 47 apoptosis-related genes were screened, including 42 up-regulated and 5 down-regulated genes during IPEC-J2 cells differentiation were identified. To determine the percentage of apoptotic cells during differentiation, conventional flow cytometry was performed using Annexin V-FITC and PI (Fig. 10B–D). Viable cells remained unstained (Annexin V-/PI-). The lower right compartment represents early apoptotic cells (Annexin V+/PI-), and the top right compartment represents late apoptotic cells (Annexin V+/PI+). Percentages are as follows: at day 1 of culture, 1.5% early and 2.86% late (Fig. 10B); at day 7 of culture, 2.71% early and 4.27% late (Fig. 10C); and at day 14 of culture, 10.52% early and 10.25% late (Fig. 10D).

## Discussion

IPEC-J2 has been established as an ideal model for studying the complex interactions occurring in the intestine. After confluence, IPEC-J2 cells undergo spontaneous differentiation that leads to the formation of a polarized monolayer, coupled by tight junctions and several morphological and functional characteristics of intestinal enterocytes [1, 8]. To determine whether proliferating and differentiated cells exhibit distinct gene expression patterns, IPEC-J2 monolayer were analyzed at transcriptional level to identify key modules and pathways involved in cell differentiation. A total of 988 differentially expressed genes (DEGs) between differentiated and undifferentiated IPEC-J2 cells were identified, including 704 up-regulated and 284 down-regulated genes. These obtained DEGs were further analyzed by GO and pathway enrichment analysis.

According to GO analysis, enriched GO terms were mainly involved in cell proliferation, apoptosis, and differentiation, further confirmed by results of annexin V-FITC/PI staining and AKP activity. Apoptosis, or programmed cell death, plays a key role during the normal development of tissues and in cellular homeostasis by eliminating the damaged cells [16]. It has been shown to be essential for epithelial turnover in the intestinal epithelium [17]. In our study, 42 up-regulated and 5 down-regulated genes related to apoptosis during IPEC-J2 cells differentiation were identified. Meanwhile, annexin V-FITC/PI double staining assay showed that a remarkable increase of apoptotic cells was noted in IPEC-J2 cells after 14 days of culture. Moreover, a total of 59 differentiation-related genes were identified in this study. The activity of AKP was a recognized marker of enterocyte differentiation [18]. We found that AKP in IPEC-J2 cells was significantly increased after 14 days of culture. Further, GO terms associated with immune response were also enriched in the present study. This result was consistent with earlier findings showing that IPEC-J2 cells expressed and produces cytokines, defensins, and toll-like receptors, which could establish communication between enterocytes and the immune system [19, 20]. Additionally, desmosome, apical junction complex, cell–cell junction, and apical part of cell, were significantly enriched cellular component in the GO analysis. These data agreed well with the previous findings that junctional complexes such as tight junctions, desmosomes, and zonula adherens were observed at the apical membrane of IPEC-J2 cells by immunoblotting [8, 11]. Kinases are involved in nearly all signal transduction processes and play a pivotal role in cell differentiation [21]. In the GO molecular function (MF) analysis, kinase binding was identified as the most significantly enriched term upon IPEC-J2 differentiation.

Enrichment for KEGG, REACTOME, and CORUM pathway analysis was performed to clarify the involved biological regulation processes during IPEC-J2 cells differentiation. KEGG analysis revealed that DEGs were mainly involved in the signaling pathways associated with metabolism. Evidence has shown that metabolic pathways not only provide the cells with energy and molecular precursors for the biosynthetic needs of proliferation, but also affect cell differentiation and fate [22]. Moreover, pathways related to immune system were also identified. Consistently, REACTOME analysis also showed that most of DEGs were enriched in cytokine signaling in immune system, including IFN-α/β signaling and IL-4/13 signaling. In addition, enriched BP-SMAD complex, TNF-α/NF-kappa B signaling complex 6, and TGF-β receptor signaling pathway were identified by CORUM analysis. These findings were in line with our observation that immune response was also significantly enriched in GO analysis. Besides their role in immune responses, the IFN signaling and TNF-α/NF-kappa B signaling also participate in cell differentiation, growth, and apoptosis regulation [23, 24]. Moreover, IL-4 was reported to mediate cell growth inhibition through activation of STAT1 [25]. TGF-β/SMAD signaling has also been shown to exhibit anti-proliferative effects [26]. Together, these data indicate that modulation of multiple cytokine responses may be involved in IPEC-J2 cells differentiation and may play roles in cell growth control.

Among the identified hub genes, STAT1, AKT3, and VEGFA were the top three genes. STAT1 is a key component of IFN signaling and mediates several cellular functions, such as cell differentiation regulation and immune system activation in response to stimulation by growth factors and cytokines [27]. Du et al. [28] reported that STAT1 expression was promoted by leucine in IPEC-J2 cells to protect against virus infection. STAT1 also serves as a key modulator of cell death via activation of death-promoting genes [29]. AKT family is known to play vital role in the regulation of metabolism, cell cycle progression, cell survival and motility [30]. Of the AKT isoforms, AKT3 has been demonstrated to up-regulate the cellular levels of ROS, which activated DNA damage response and thereby inhibiting cell proliferation [31]. VEGFA is a biologically active peptide essential for angiogenesis and participates in the control of cell growth, differentiation, adhesion, wound healing and tissue repair [32, 33]. VEGFA can promote intestinal cell migration and repair of intestinal epithelial cell damage [34], and may be implicated in pathogen-induced IPEC-J2 cell injury [35]. Based on these studies, we speculate that these three genes may be important for IPEC-J2 cells differentiation. However, in this study, the expression profiles of differentiated IPEC-J2 cells were investigated at the transcriptional level. Given that gene expression does not necessarily translate into production and secretion of proteins, more laboratory work is needed to validate these results.

## Conclusions

In conclusion, regulation of cell population, apoptotic signaling pathway, epithelial cell differentiation, cell junctions, and cytokine responses regulation were mainly identified during IPEC-J2 cells differentiation. Moreover, STAT1, AKT3, and VEGFA genes were also involved in IPEC-J2 differentiation. These findings of this study may contribute to the molecular characterization of IPEC-J2 cells as a model to study swine intestinal physiology, and may progress the understanding of intestinal development and cellular differentiation of pig intestinal epithelium.

## Data Availability

All data will be available from the corresponding author upon reasonable request.

## References

[1] Vergauwen H. The IPEC-J2 cell line: the impact of food bioactives on health: in vitro and ex vivo models. In: Verhoeckx K, Cotter P, López-Expósito I, Kleiveland C, Lea T, Mackie A, Requena T, Swiatecka D, Wichers H, editors. Cham (CH): Springer; 2015. p. 125–34. 29787039

[2] Saleri R, Borghetti P, Ravanetti F, Andrani M, Cavalli V, De Angelis E, Ferrari L, Martelli P (2021). A co-culture model of IPEC-J2 and swine PBMC to study the responsiveness of intestinal epithelial cells: the regulatory effect of arginine deprivation. Animals (Basel).

[3] Yang Y, Huang J, Li J, Yang H, Yin Y (2020). The Effects of butyric acid on the differentiation, proliferation, apoptosis, and autophagy of IPEC-J2 Cells. Curr Mol Med.

[4] Bernardini C, Algieri C, La Mantia D, Trombetti F, Pagliarani A, Forni M, Nesci S (2021). Vitamin K vitamers differently affect energy metabolism in IPEC-J2 Cells. Front Mol Biosci.

[5] Tang J, Cao L, Jia G, Liu G, Chen X, Tian G, Cai J, Shang H, Zhao H (2019). The protective effect of selenium from heat stress-induced porcine small intestinal epithelial cell line (IPEC-J2) injury is associated with regulation expression of selenoproteins. Br J Nutr.

[6] Vergauwen H, Tambuyzer B, Jennes K, Degroote J, Wang W, De Smet S, Michiels J, Van Ginneken C (2015). Trolox and ascorbic acid reduce direct and indirect oxidative stress in the IPEC-J2 cells, an in vitro model for the porcine gastrointestinal tract. PLoS ONE.

[7] Zhuang Y, Wu H, Wang X, He J, He S, Yin Y (2019). Resveratrol attenuates oxidative stress-induced intestinal barrier injury through PI3K/Akt-mediated Nrf2 signaling pathway. Oxid Med Cell Longev.

[8] Schierack P, Nordhoff M, Pollmann M, Weyrauch KD, Amasheh S, Lodemann U, Jores J, Tachu B, Kleta S, Blikslager A (2006). Characterization of a porcine intestinal epithelial cell line for in vitro studies of microbial pathogenesis in swine. Histochem Cell Biol.

[9] Brosnahan AJ, Brown DR (2012). Porcine IPEC-J2 intestinal epithelial cells in microbiological investigations. Vet Microbiol.

[10] Hussain T, Yuan D, Tan B, Murtaza G, Rahu N, Kalhoro MS, Kalhoro DH, Yin Y (2020). Eucommia ulmoides flavones (EUF) abrogated enterocyte damage induced by LPS involved in NF-κB signaling pathway. Toxicol In Vitro.

[11] Zakrzewski SS, Richter JF, Krug SM, Jebautzke B, Lee IF, Rieger J, Sachtleben M, Bondzio A, Schulzke JD, Fromm M (2013). Improved cell line IPEC-J2, characterized as a model for porcine jejunal epithelium. PLoS ONE.

[12] Yeung TM, Chia LA, Kosinski CM, Kuo CJ (2011). Regulation of self-renewal and differentiation by the intestinal stem cell niche. Cell Mol Life Sci.

[13] Wang Q, Zhou Y, Rychahou P, Fan TW, Lane AN, Weiss HL, Evers BM (2017). Ketogenesis contributes to intestinal cell differentiation. Cell Death Differ.

[14] Geens MM, Niewold TA (2011). Optimizing culture conditions of a porcine epithelial cell line IPEC-J2 through a histological and physiological characterization. Cytotechnology.

[15] Zhou Y, Zhou B, Pache L, Chang M, Khodabakhshi AH, Tanaseichuk O, Benner C, Chanda SK (2019). Metascape provides a biologist-oriented resource for the analysis of systems-level datasets. Nat Commun.

[16] Laghezza Masci V, Ovidi E, Taddei AR, Turchetti G, Tiezzi A, Giacomello P, Garzoli S. Apoptotic effects on HL60 human leukaemia cells induced by lavandin essential oil treatment. Molecules. 2020; 25. 10.3390/molecules25030538 PMC7036901 31991893

[17] Günther C, Neumann H, Neurath MF, Becker C (2013). Apoptosis, necrosis and necroptosis: cell death regulation in the intestinal epithelium. Gut.

[18] Budd GR, Aitchison A, Day AS, Keenan JI (2017). The effect of polymeric formula on enterocyte differentiation. Innate Immun.

[19] Burkey TE, Skjolaas KA, Dritz SS, Minton JE (2009). Expression of porcine Toll-like receptor 2, 4 and 9 gene transcripts in the presence of lipopolysaccharide and Salmonella enterica serovars Typhimurium and Choleraesuis. Vet Immunol Immunopathol.

[20] Arce C, Ramírez-Boo M, Lucena C, Garrido JJ (2010). Innate immune activation of swine intestinal epithelial cell lines (IPEC-J2 and IPI-2I) in response to LPS from Salmonella typhimurium. Comp Immunol Microbiol Infect Dis.

[21] Chang TL, Peng X, Fu XY (2000). Interleukin-4 mediates cell growth inhibition through activation of Stat1. J Biol Chem.

[22] Stafford S, Lowell C, Sur S, Alam R (2002). Lyn tyrosine kinase is important for IL-5-stimulated eosinophil differentiation. J Immunol.

[23] Agathocleous M, Harris WA (2013). Metabolism in physiological cell proliferation and differentiation. Trends Cell Biol.

[24] Kalvakolanu DV (2000). Interferons and cell growth control. Histol Histopathol.

[25] Fish EN, Platanias LC (2014). Interferon receptor signaling in malignancy: a network of cellular pathways defining biological outcomes. Mol Cancer Res.

[26] Jung B, Staudacher JJ, Beauchamp D (2017). Transforming growth factor β superfamily signaling in development of colorectal cancer. Gastroenterology.

[27] Du J, Chen D, Yu B, He J, Yu J, Mao X, Luo Y, Zheng P, Luo J (2021). L-Leucine promotes STAT1 and ISGs expression in TGEV-infected IPEC-J2 cells via mTOR activation. Front Immunol.

[28] Kim HS, Lee MS (2007). STAT1 as a key modulator of cell death. Cell Signal.

[29] Zhang Y, Liu Z (2017). STAT1 in cancer: friend or foe?. Discov Med.

[30] Hua H, Zhang H, Chen J, Wang J, Liu J, Jiang Y (2021). Targeting Akt in cancer for precision therapy. J Hematol Oncol.

[31] Polytarchou C, Hatziapostolou M, Yau TO, Christodoulou N, Hinds PW, Kottakis F, Sanidas I, Tsichlis PN (2020). Akt3 induces oxidative stress and DNA damage by activating the NADPH oxidase via phosphorylation of p47(phox). Proc Natl Acad Sci USA.

[32] Drucker DJ (2003). Glucagon-like peptides: regulators of cell proliferation, differentiation, and apoptosis. Mol Endocrinol.

[33] Kitamura T, Asai N, Enomoto A, Maeda K, Kato T, Ishida M, Jiang P, Watanabe T, Usukura J, Kondo T (2008). Regulation of VEGF-mediated angiogenesis by the Akt/PKB substrate Girdin. Nat Cell Biol.

[34] Bulut K, Pennartz C, Felderbauer P, Ansorge N, Banasch M, Schmitz F, Schmidt WE, Hoffmann P (2006). Vascular endothelial growth factor (VEGF164) ameliorates intestinal epithelial injury in vitro in IEC-18 and Caco-2 monolayers via induction of TGF-beta release from epithelial cells. Scand J Gastroenterol.

[35] Luo R, Yan Z, Yang Q, Huang X, Gao X, Wang P, Wang W, Xie K, Gun S (2020). Inhibition of ssc-microRNA-140-5p ameliorates the Clostridium perfringens beta2 toxin-induced inflammatory response in IPEC-J2 cells via the ERK1/2 and JNK pathways by targeting VEGFA. Mol Immunol.

